# Expression Stabilities of Candidate Reference Genes for RT-qPCR under Different Stress Conditions in Soybean

**DOI:** 10.1371/journal.pone.0075271

**Published:** 2013-10-04

**Authors:** Shuhua Ma, Hongwei Niu, Chunji Liu, Jie Zhang, Chunyan Hou, Dongmei Wang

**Affiliations:** 1 College of Life Science, Agricultural University of Hebei, Baoding, Hebei Province, China; 2 CSIRO Plant Industry, Queensland Bioscience Precinct, Australia; Queen’s University Belfast, United Kingdom

## Abstract

Due to its accuracy, sensitivity and high throughput, real time quantitative PCR (RT-qPCR) has been widely used in analysing gene expression. The quality of data from such analyses is affected by the quality of reference genes used. Expression stabilities for nine candidate reference genes widely used in soybean were evaluated under different stresses in this study. Our results showed that *EF1A* and *ACT11* were the best under salinity stress, *TUB4*, *TUA5* and *EF1A* were the best under drought stress, *ACT11* and *UKN2* were the best under dark treatment, and *EF1B* and *UKN2* were the best under virus infection. *EF1B* and *UKN2* were the top two genes which can be reliably used in all of the stress conditions assessed.

## Introduction

Due to its accurate quantification, high sensitivity and high throughput, real time quantitative PCR (RT-qPCR) has been widely used in analyzing gene expression, in determining number of gene copies and in detecting the presence of microbes in food products [1]. Reference genes are required in RT-qPCR analysis to minimize influences of RNA quality and quantity and efficiency of reverse transcription [2], [3].

House-keeping genes are often selected as reference genes. The most commonly used reference genes include β-actin (*ACT*), glyceral-dehyde-3-phosphate dehydrogenase (*GAPDH*), 18S ribosomal RNA (*18S rRNA*), 25S ribosomal RNA (*25S rRNA*), polyubiquitin (*UBQ*), ubiquitin conjugating enzyme (*UBC*), translation elongation factor (*TEF*), cyclophylin (*CYP*), elongation factor 1-A (*EF1A*) and tubulin (*TUB*) etc. [3]–[5]. Ideal reference genes should provide stable expression in different plant tissues, at different stages of development or under different environments of experiments. However, it has been reported that many of the house-keeping genes provide stable expression under only certain environments and it is necessary to identify and select suitable reference genes for a given experiment [6]. There are also many reports showing that the use of 2 or 3 reference genes could be necessary as using a single reference gene could lead to significant error [7], [8]. Efforts in identifying suitable reference genes have been reported in many crop species including *Arabidopsis thaliana* [9]–[11], *Brassica napus* [12], soybean [13], *Pisum sativum* [14], rice [5], [15]–[16], *Platycladus orientalis* [17], coffee [18], *Gossypium hirsutum* [19], tomato [20], [21] and water lily [22]. Several new reference genes have been identified [23]–[25]. It has been reported that *SKIP16* (SKIP/Ask-Interacting Protein 16), *MTP* (Metallo protease, Insulin degrading Enzyme), *UKN1* and *UKN2* (Hypothetical protein) all gave stable expressions at different stages of development in soybean [13]. Investigating mechanisms of viral resistance in soybean has been one of our research foci. We have demonstrated that callose deposition at the plasmodesmata plays a critical role in host resistance to viral infection [26]. During the investigation of the physiological mechanism of callose deposition, we analysed the expression of callose synthase and β-1, 3-glucanase using RT-qPCR and observed that the expression levels of these genes were different with the use of different reference genes (data not shown). Based on this observation, we systematically investigated expression stabilities of eight widely used (including *ACT11, TUA5, CYP, EF1B, TUA4, TUB4, EF1A* and *ACT2/7*) and one recently reported (*UKN2*) reference genes under viral infection and stresses of darking, salinity and drought, with the objective of identifying those which provide stable expressions in each of the environments assessed.

## Materials and Methods

### Plant Genotypes

Two soybean (*Glycine max* (L.) Merrill) cultivars Jidou 7 and Nannong 1138-2 were used in this study. The former was used for various treatments as described below and the latter was used for preparing inoculums of *soybean mosaic virus* (SMV). Seeds were germinated and grown in a greenhouse with a 14 h light/10 h dark cycle at a constant temperature of 25°C and 700 µmol photons m^−2^ s^−1^.

### Experiments Conducted

Following experiments were conducted when the unifoliate leaves of the seedlings were fully unfolded:

Infection of the soybean seedlings with SMV: The SMV inoculums were prepared by grinding leaves of infected soybean cv. Nannong 1138-2 to slurry with a pestle in a mortar with 0.1 M phosphate buffer, pH 7.4. Carborundum was used as an abrasive. The unifoliate leaves of cv. Jidou 7 were inoculated by rubbing with a brush. The leaves were rinsed with distilled water immediately after inoculation. Leaf samples were taken at 0, 8, 24, and 48 hours post inoculation.

Dark treatment: the seedlings were transferred in to a box for this experiment. Leaf samples were taken at 0, 2, 4, and 6 hours respectively post the treatment.

Salt treatment: This experiment was conducted hydroponically. The seedlings were transferred to a complete nutrient solution containing 200 mM NaCl. Leaf samples were taken at 0, 2, 16, 20 hours post treatment.

Drought treatment: This experiment was also conducted hydroponically. The seedlings were transferred to a complete nutrient solution containing 15% PEG_6000_. Leaf samples were taken at 0, 2, 4, and 6 hours post treatment.

### RNA Isolation and cDNA Synthesis

RNA was extracted using RNAiso Plus (TaKaRa, Japan) according to the manufacturer’s instructions, and genomic DNA was then eliminated using RNase-free DNase I (TaKaRa, Japan). The quantity and quality of the total RNA extracted was determined using a Nanophotometer p-class K5600. Only RNA samples meeting the following requirements were used in this study: Firstly, they should have an absorbance ratio at OD260/280 between 1.8 and 2.2; secondly, their absorbance ratio at OD260/230 should be about 2.0; thirdly, the ratio of 28S/18S ribosomal RNA should be between 1.5 and 2.0; and fourthly, they should have little smears on agarose gels. First strand cDNA was synthesized by reverse transcribing 500 ng of total RNA with PrimeScript®RT reagent Kit (Perfect Real Time) (TaKaRa, Japan). All cDNA were stored at −20°C until use.

### Primer Design and RT-qPCR

Primers for the 9 reference genes were designed using Primer 3 software (Table 1). These primer sets have a single melting temperature of 55°C and the amplicons amplified by them vary between 100–200 bp in length. The primers were synthesized by Shanghai Biological Engineering Technology Services Company.

**Table 1:** Primer sequences and related information for each candidate reference gene.

Genesymbol	Genename	NCBIAccessionNo.	Arabidopsishomologlocus	Primer sequence (5′–3′)		Size(bp)	Function
*ACT11*	Actin 11	LOC100781831	AT3G12110	ATTTTGACTGAGCGTGGTTATTCC	GCTGGTCCTGGCTGTCTCC	126	Cytoskeletalstructuralprotein
*TUA5*	AlphaTubulin	LOC732582	AT5G19780	TGCCACCATCAAGACTAAGAGG	ACCACCAGGAACAACAGAAGG	103	Structuralconstituent ofcytoskeleton
*CYP*	Cyclophilin	LOC100500498	AT2G21130	ACGACGAAGACGGAGTGG	CGACGACGACAGGCTTGG	130	Proteinfolding
*EF1B*	Elongationfactor1-beta	LOC100500082	AT5G12110	CCACTGCTGAAGAAGATGATGATG	AAGGACAGAAGACTTGCCACTC	134	Translationalelongation
*TUA4*	alphaTubulin	LOC100781185	AT1G50010	CATACCCTAGAATCCATTTC	TGTACTTTCCGTGACGAG	156	Structuralconstituent ofcytoskeleton
*TUB4*	betaTubulin	LOC100798849	AT5G12250	TGGCGTCCACATTCATTG	GAACTCCATCTCGTCCAT	137	Structuralconstituent ofcytoskeleton
*EF1A*	Eukaryoticelongationfactor1-alpha	LOC100785429	AT5G60390	GACCTTCTTCGTTTCTCGCA	CGAACCTCTCAATCACACGC	162	Translationalelongation
*ACT2/7*	Actin2/7	LOC100789000	AT5G09810	CTTCCCTCAGCACCTTCCAA	GGTCCAGCTTTCACACTCCAT	119	Cytoskeletalstructuralprotein
*UKN2*	Hypotheticalprotein	LOC100789577	AT4G33380	TGTGCTCTGTGAAGAGATTG	TCATAATCTGTGTGCAGTTC	156	Unkown

RT-qPCR reactions were carried out in 96-well blocks with an Applied CHROMO4 Real-Time PCR system using SYBR premix Ex Taq II kit (TaKaRa, Japan). After 40 cycles, a melting curve analysis was carried out (60°C to 95°C) to verify the specificity of amplicons. Each amplification was repeated 3 times. The specificity of the amplicons was confirmed by the presence of a single peak.

### Statistical Analysis

The relative level of expression (Q) for a given gene was calculated based on the formula Q = 2^−△Ct^. Each Ct value represents the average of three replicates. △Ct equals to Ct_samples_–Ct_min_ (Ct_min_ being the one with the lowest Ct value in all samples and Ct_samples_ represents each sample by Ct value) [15].

For selecting the appropriate reference genes, we used GeNorm [27] and NormFinder [28] in analysing the expression stability of the genes assessed. The Q value was imported into GeNormv3.5 and NormFinder for reference gene selection. Two parameters were obtained when Q values were loaded into GeNorm, an average expression stability value (M value) and an average pairwise variation of template normalization factor (V_n/n+1_ value). The M value of a gene was inversely correlated with its expression stability. The default setting for the cut-off value V (0.15) was used. Thus, if V_n/n+1_≤0. 15, it is not necessary to introduce n+1 reference genes as the internal control [6]. Therefore, through these two parameters we can analyze the most stable reference genes and obtain the required reference gene number.

Similar to GeNorm, NormFinder also generates Q from the original data via either standard curve (Figure S3) or ΔCt method and then use the software to obtain gene expression stable value M through analysis of variance and direct assessment of genetic stability.

With purified PCR product as a starting template, followed by five times of 10-fold serial dilutions (10^0^ (PCR product 500 times dilution), 10^−1^, 10^−2^, 10^−3^, 10^−4^, respectively) creating a gradient of concentrations. Standard curve was drawn according to the results of RT-qPCR (Figure S3). The formula E = (10^−1/slope^−1)×100 was used to calculate the amplification efficiencies of the genes and the calculation results were shown in Table 2. Primer amplification efficiency between 95%–105% can be used.

**Table 2:** Efficiency of designed primer pairs used for RT-qPCR amplification.

Gene symbol	Arabidopsis homolog locus	Tm (°C)	PCR efficiency (%)	Regression coefficient (R2)
*ACT11*	AT3G12110	82.82	103.89	0.999
*TUA5*	AT5G19780	80.97	103.89	0.998
*CYP*	AT2G21130	87.23	97.65	0.996
*EF1B*	AT5G12110	82.05	110.77	0.999
*TUA4*	AT1G50010	83.28	106.78	0.986
*TUB4*	AT5G12250	83.66	100.59	0.998
*EF1A*	AT5G60390	81.97	105.68	0.998
*ACT2/7*	AT5G09810	81.15	99.99	0.995
*UKN2*	AT4G33380	83.98	100.59	0.997

## Results

### Efficiency and Specificity of Designed Primer Pairs Used for RT-qPCR Amplification

The specificity of each primer pair can be assessed by RT-qPCR analysis. Figure S1 showed that each melting curve of the 9 reference genes assessed was characterized as a single peak (Figure S1), indicating that the primer pairs used are highly specific. The specificity of these primer pairs was also confirmed by running a 2% agarose gel (Figure S2).

Accurate analysis of RT-qPCR data requires that all primer pairs used should have the same amplification efficiency. The rate of amplification efficiency at 100% or close to it indicates that the reaction conditions are optimal and the results obtained should be highly repeatable. Experimentally, the amplification efficiency of each primer pair should be between 95–105%, thus it can be used for the next step of experiments. The amplification efficiencies for the nine primer pairs used in this study were shown in Table 2, and they were all between 97.65–110.77%. Correlation coefficients (R^2^) were all larger than 0.98, indicating that all of the primer pairs are highly specific and efficient in RT-qPCR amplification.

### Expression Levels of the Candidate Reference Genes Assessed

**Figure 1:**
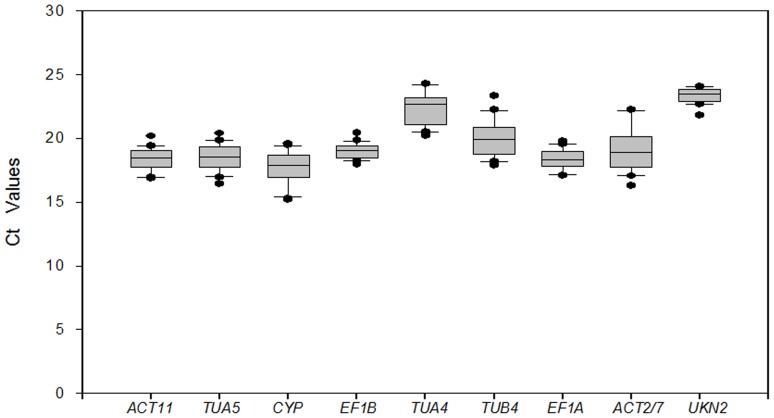
Candidate reference gene expression levels in different samples. Expression data displayed as Ct values for each reference gene in all samples. A line across the box is depicted as the median. The box indicates the 25th and 75th percentiles. Whiskers represent the maximum and minimum values.

Ct value represents the number of cycles that the PCR product appear to effectively increase [29], [30]. By calculating Ct value, we can compare the mRNA abundance of each of the tested genes. The higher the Ct value, the lower the mRNA level of the gene; Conversely, the lower the Ct value, the higher the mRNA level of the gene. Average Ct values for most of the nine reference genes under different treatments varied between 18 and 20, whereas the full range varied between 18 and 25 (Figure 1). The expression levels of *TUA4* and *UKN2* were lower than those of the other 7 genes, and their Ct values were 22 and 24, respectively. The Ct values of the reference genes were variable under different treatment conditions. The differences of Ct values (coefficient of variation) indicate the stability of the reference gene expression, the greater the coefficient of variation of the gene, the more unstable of the gene expression. The coefficient of variation of *ACT2/7* was high above six cycles and those of *EF1B* and *UKN2* were low (approximately two cycles). Calculating the Ct value for determining the reference gene expression stability in different samples is important to select the internal reference standard.

### GeNorm Analysis

**Figure 2:**
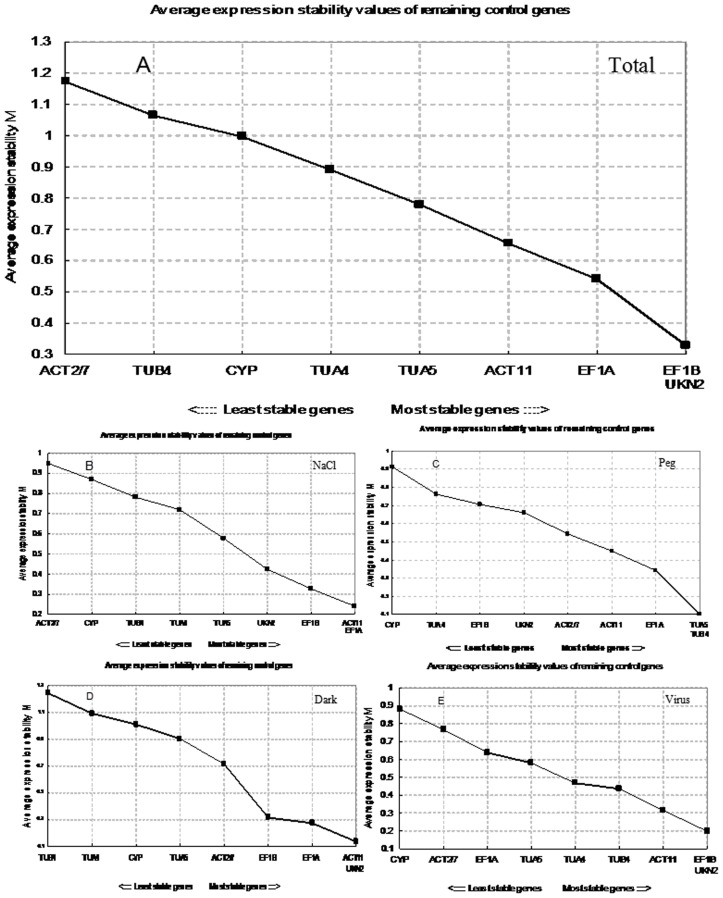
Gene expression stability and ranking of 9 reference genes as calculated by GeNorm. Expression stability and ranking of 9 reference genes calculated with GeNorm in all samples (A), NaCl-treated (B), PEG-treated (C), Dark-treated (D), Virus-treated (E); A lower average expression stability M value indicates more stable expression.

GeNorm software can analyze and determine the most stable reference gene by analysising the stability of the reference gene expression (M value) in different samples. The default value suggested by GeNorm is M = 1.5, the reference gene, of which the M value is less than 1.5, is considered to be used as an internal reference gene. The larger the M value, the worse the stability. Figure 2A shows the results of the analysis of all 16 samples under the four stress conditions. As shown, *EF1B* and *UKN2* gave the lowest M values (M = 0.328), indicating that the expression stability of the two reference genes were the best among the nine tested genes. *ACT2/7* showed the highest M value (M = 1.172), indicating that it was the most unstable gene.

The stabilities of the nine tested reference genes were not consistent under different treatment conditions. Under salt treatment, the most stable genes were *ACT11* and *EF1A* and the most unstable one was *ACT2/7* (Figure 2B); under drought treatment, the most stably expressed genes were *TUA5* and *TUB4* and the most unstable one was *CYP* (Figure 2C); under dark treatment, *ACT11* and *UKN2* were the most stably expressed genes while *TUB4* was the most unstable one (Figure 2D); under virus stress, *EF1B* and *UKN2* were the most stably expressed genes while *CYP* the most unstably expressed one (Figure 2E). The M values for all of these tested reference genes were all lower than the default value of M = 1.5.

**Figure 3:**
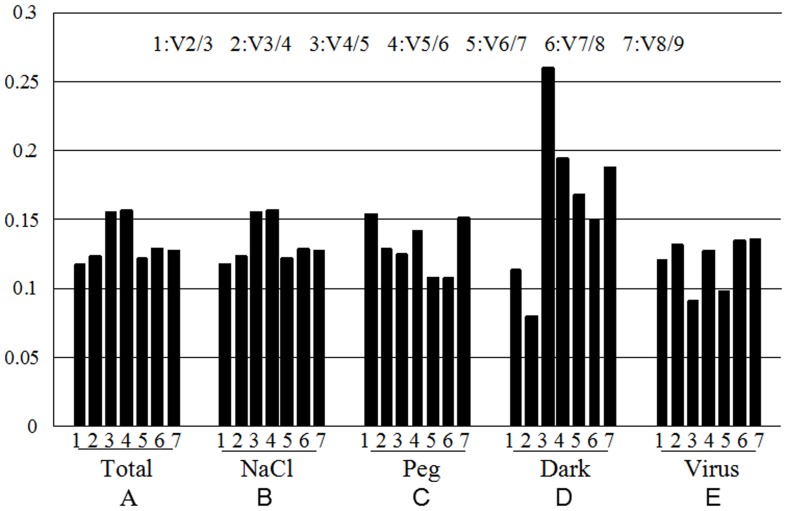
Determination of the optimal number of reference genes for normalization by pairwise variation (V) using GeNorm. The pairwise variation(V) to determine the optimal number of reference gene for accurate normalization in all samples (A), NaCl-treated (B), PEG-treated (C), Dark-treated (D), Virus-treated (E). It is the representative of the V2/3, V3/4, V4/5, V5/6, V6/7, V7/8, V8/9 form one to seven.

In order to get more reliable results from RT-qPCR, it is generally recommended to use two or more reference genes. GeNorm software could analyze the pairwise variation value of the normalization factor (V) [19]. By using the V factor, the appropriate number of reference genes could be determined under different treatments. Therefore, It is proposed that 0.15 could be used as the cut-off value for V, below which the inclusion of an additional control gene is not required; that is, if V_n/n+1_<0.15, it is not necessary to use n+1 reference genes as internal controls. The analysis results were shown in Figure 3, under the salt stress (Figure 3B) and viral infection (Figure 3E), the V_2/3_<0.15, the third reference gene failed to significantly expressed standardization factor differences, indicating that under these two treatments chosen the two most stable expressed genes, *EF1A* and the *ACT11(* Figure 2B *)*, and *EF1B* and *UKN2* (Figure 2E), were enough to be used as accurate standardized controls. Under the drought treatment (Figure 3C), V_2/3_>0.15, it was necessary to introduce a third reference gene, which means that the introduction of the third gene could enable a smaller V value. This was proved to be true by the analysis result, V_3/4_<0. 15, therefore the three genes, *TUB4*, *TUA5* and *EF1A* were chosen as reference genes under this condition (Fig. 2C), which suggested that group cooperation of internal references can get more accurate data. Under dark treatment (Figure 3D), although the value of V_3/4_ is smaller, but V_2/3_<0.15, therefore *ACT11* and *UKN2* (Figure 2D) would be sufficient used as cooperation internal references as accurately standardized controls. Overall analysis of all the samples, V_2/3_ and V_3/4_ values were less than 0.15 (Figure3E), which indicated that *EF1B* and *UKN2* can be used as the standardization of gene expressions under various stress treatments in this study (Figure 2E).

### NormFinder Analysis

In this study, NormFinder software was also used to determine the best reference gene as a standardization of RT-qPCR. Like GeNorm software, lower M values indicate the higher stability of a given gene. Results obtained through NormFinder software are shown in Table 3.

**Table 3:** Ranking of candidate reference genes in order of their expression stability as calculated by NormFinder.

	Total(A)	NaCl(B)	Peg(C)	Dark(D)	Virus(E)
1	*ACT11*	*EF1A*	*TUB4*	*ACT2/7*	*TUB4*
M value	0.436	0.210	0.092	0.327	0.096
2	*EF1A*	*EF1B*	*ACT11*	*CYP*	*TUA4*
M value	0.436	0.269	0.139	0.518	0.212
3	*TUA4*	*ACT11*	*EF1A*	*EF1B*	*UKN2*
M value	0.475	0.325	0.200	0.528	0.301
4	*TUA5*	*UKN2*	*TUA5*	*EF1A*	*EF1B*
M value	0.495	0.427	0.210	0.534	0.322
5	*EF1B*	*TUA5*	*ACT2/7*	*TUA5*	*TUA5*
M value	0.505	0.435	0.452	0.594	0.349
6	*UKN2*	*TUA4*	*UKN2*	*TUA4*	*ACT11*
M value	0.506	0.494	0.551	0.693	0.357
7	*TUB4*	*TUB4*	*EF1B*	*UKN2*	*EF1A*
M value	0.653	0.525	0.583	0.698	0.454
8	*CYP*	*CYP*	*TUA4*	*ACT11*	*ACT2/7*
M value	0.742	0.749	0.594	0.728	0.795
9	*ACT2/7*	*ACT2/7*	*CYP*	*TUB4*	*CYP*
M value	0.950	0.777	0.935	1.155	0.835

From Table 3, we found that the most stably expressed genes in all samples were *ACT11* and *EF1A*, the same as those obtained by GeNorm software, and *ACT2/7* was calculated to be the most unstable expressed gene. Under salt treatment, *EF1A* and *EF1B* had the lowest value of M indicating that the two genes were the most stably expressed ones, and *ACT2/7* and *CYP* were the most unstable ones. Under drought treatment and virus infection, *TUB4* was the most stable expressed gene and *CYP* was the most unstable one. Under dark treatment, the most stably expressed genes were *ACT2/7* and *CYP*. Their M values were 0.327 and 0.518, respectively. *TUB4* was the most unstably expressed gene under this treatment. Overall, the results obtained through the two GeNorm and NormFinder softwares were not very consistent. It is possible that the inconsistent is due to the different calculation methods used between the two softwares.

## Discussion

The selection of suitable reference genes is critical in obtaining accurate results from RT-qPCR analysis. The expression stabilities of nine of the possible candidate genes, including *ACT11*, *TUA5, CYP*, *EF1B*, *TUA4*, *TUB4*, *EF1A*, *ACT2/7* and *UKN2*, were investigated under different stress conditions in a study reported in this paper. Our results showed that the most stably expressed genes were *EF1A* and *ACT11* under salinity treatment, were *TUB4*, *TUA5* and *EF1A* under drought treatment, were *ACT11* and *UKN2* under dark treatment, and were *EF1B* and *UKN2* under virus infection. Overall *EF1B* and *UKN2* gave the best expression stabilities under each of the four treatments assessed.

Studying its expression is a critical component in determining the function of a given gene in molecular biology and such studies help us to understand the growth and development of different species. RT-qPCR is the most commonly used method in such studies and the use of appropriate reference genes is essential in accurately determining the expression quantity of a given gene [31]. Ideal reference genes are those which give constant expression levels. However, such genes may not exist as plant growth is affected by environments. Different results can be obtained with the use of different reference genes and inaccurate assessment of gene expression could be obtained if suitable reference genes are not used. For example, Mafra et al. analysed the relative expression levels of *WRKY70* (transcription factor) in citrus challenged with fungal pathogens and found that, when GeNorm was used, the use of the two most variable reference genes (*CYP* and *TUB*) resulted in an 42 folds increase in the relative transcript abundance of this gene [32]. However, the use of the three most stable reference genes (*DIM1*, *GAPC2* and *PTB1*) resulted in only a four folds increase in the relative transcript abundance of this gene. Similar results were also obtained by Le et al. in soybean shoots [33]. When *SUB12*, *60 s* and *Fbox* were used as reference genes, the relative transcript abundance of *GmNAC19* was 1.12, 18.66 and 13.93, respectively, when samples were taken at 10 h post dehydration stress. In an analysis of β-1,3-glucanase BG1 in soybean leaves following virus infection, we found that the relative transcript abundance of this gene was different with the use of different reference genes and that its abundance also varied at different times of post inoculation(unpublished). When *TUA4* was used as the reference gene, the relative transcript abundance of *BG1* peaked at 48 h post inoculation (76 folds). However, when the most stable reference gene *UKN2* identified in the study was used, the relative abundance of *BG1* did not peak until 144 h post inoculation when its abundance was 36.7 folds of that of the control (data unpublished). These data showed that the selection of suitable reference genes is critical in RT-qPCR analysis.

In the study, nine candidate reference genes (eight of them have been widely used) were analysed under four biotic and abiotic stresses. Results obtained from analysing the RT-qPCR data by using the GeNorm software showed that *UKN2* and *EF1B* were the most stably expressed reference genes, which was followed by *EF1A*. However, the most stably expressed reference gene was *ACT11*, which was followed by *EF1A* and *TUA4*, when the data were analyzed by using the NormFinder software. Both methods found that *ACT2/7* was the most unstable gene, thus we should avoid to use it as the reference gene in determining the expression quantity of a given gene through RT-qPCR analysis in soybean.

Samples from different stress treatments were also analysed in this study. The results showed that *EF1A* and *TUB4* were the most stably expressed genes under stresses of salinity and water, and *ACT2/7* and *CYP* were the most unstable ones under these conditions (Fig. 2; Table 3). Different results were obtained when the dark treatment data were analyzed using the two different softwares. The most stable gene was *ACT2/7* when the data were analysed by NormFinder. However it was *UKN2* when GeNorm was used, although *TUB4* was found to be the most unstable gene using either of the two softwares.

Similarly, different results were also obtained in analyzing the data from the experiments of viral infection using the two different softwares, GeNorm and NormFinder. The most stable gene was *TUB4* when the data were analyzed by NormFinder, but it was changed to be *UKN2* when GeNorm was used, although *CYP* was found to be the most unstable gene using either of the two softwares (Fig. 2; Table 3). These results showed that the nine candidate genes have different expression stabilities under different conditions of stresses, and using the two different softwares may lead to different selection of the best reference genes. Thus it is very important to selectively exploit different reference genes for different experiments by combing two or more software analyses.

There have numerous reports on the identification of suitable reference genes for RT-qPCR analysis. Jian [34] reported a study in which ten of the most widely used reference genes were analyzed using GeNorm. It was found that *EF1B* and *CYP2* were the most stable ones among all the samples examined; that *EF1B* and *CYP2* were the most stable ones across the different tissues at a given stage of plant development, that *ACT2/7* and *TUA* were the best ones across the different stages of plant development, and that *ACT11* and *EF1B* were the best ones across the different lighting periods examined. These results were slightly different from what we obtained in this paper, which is very likely due to the different materials and/or stress conditions used.

Hu et al. (2009) studied the expression stability of 14 reference genes under different conditions. Using GeNorm and NormFinder softwares, they found that *SKIP16*, *UKN1* and *UKN2* were the most stable ones among all of the samples examined [13]. Considering the fact that *UKN2* was also the best reference gene in our study, it is highly recommended that *UKN2* could be safely used for RT-qPCR analysis as a reference gene. There are many reports claiming that the expression stability of *ACT2/7* was poor. However, its expression stability was good in different tissues of *Arabidopsis thaliana* and *Platycladus orientalis* [17] and under low-temperature (cold) stress.

Ideal reference genes are those which give constant expression levels. However, such genes may not exist as plant growth is affected by environments. Different results can be obtained with the use of different reference genes and inaccurate assessment of gene expression could be resulted if suitable reference genes are not used. In this study, we investigated the expression stabilities of nine candidate genes under different stress conditions. Our results showed that the expression of each of these genes varied to some degree among the different treatments and none of them fits the definition of house-keeping genes. In order to get reliable results in gene expression in soybean, two or more reference genes need to be used in RT-qPCR analysis. In addition, we also showed that similar results were obtained from using both GeNorm and NormFinder softwares, and the top four reference genes detected among all of the samples by these two softwares were similar. These results demonstrate that the reference genes selected from this study were reliable and they form a solid base for conducting functional gene expression analysis in soybean.

## Supporting Information

Figure S1 **Dissociation curve data for the 9 reference genes.**
(TIF)

Figure S2 **RT-qPCR amplification specificity of the 9 reference genes.** Amplification fragments were separated by 2% agarose gel electrophoresis.
(TIF)

Figure S3 **RT-qPCR standard curve of the 9 reference genes.**
(TIF)
