# Supplementary figures and images for: Expression Stabilities of Candidate Reference Genes for RT-qPCR under Different Stress Conditions in Soybean
Source: PLoS One. 2013 Oct 4;8(10):e75271. doi: 10.1371/journal.pone.0075271 (PMC3790784; doi:10.1371/journal.pone.0075271)

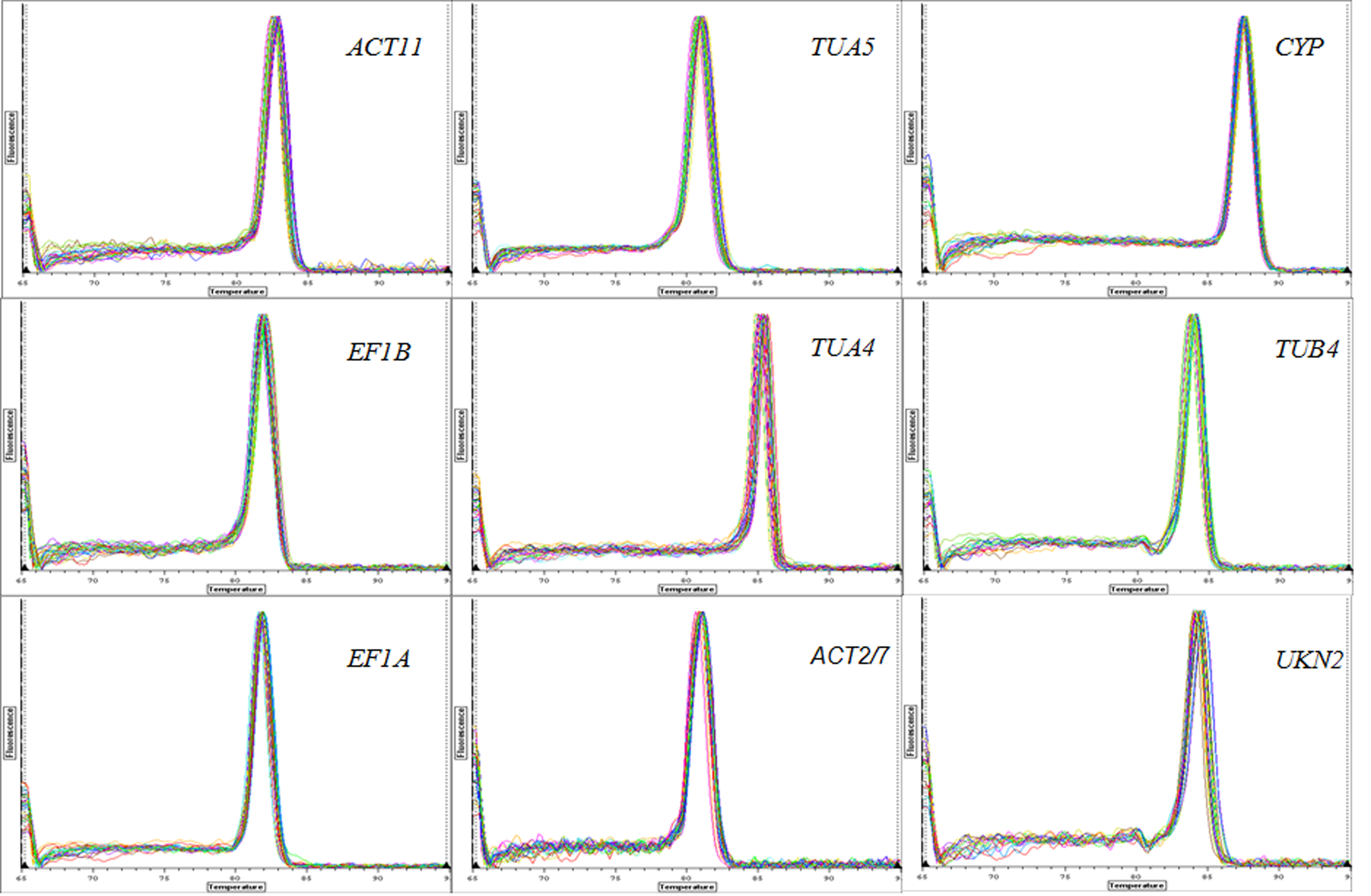

Supplement: Figure S1 — Dissociation curve data for the 9 reference genes. (TIF) [file pone.0075271.s001.tif]

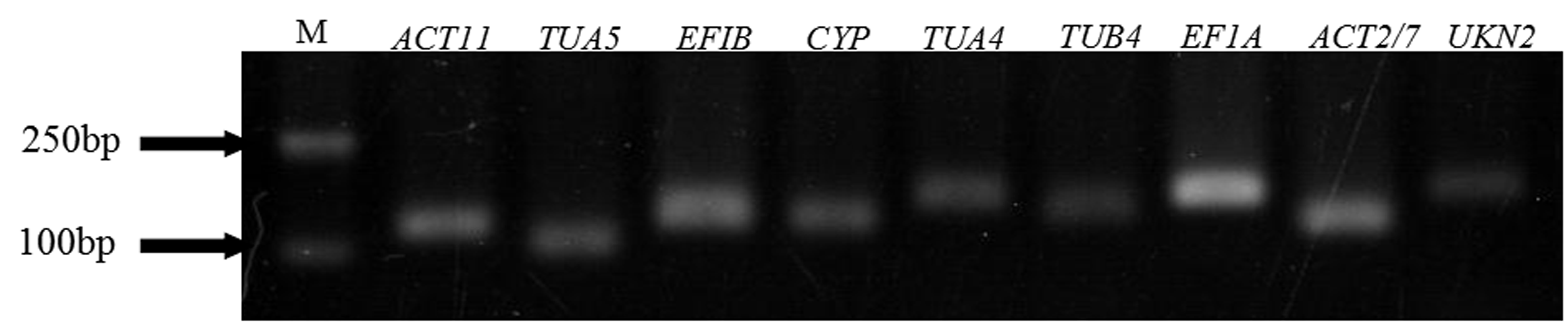

Supplement: Figure S2 — RT-qPCR amplification specificity of the 9 reference genes. Amplification fragments were separated by 2% agarose gel electrophoresis. (TIF) [file pone.0075271.s002.tif]

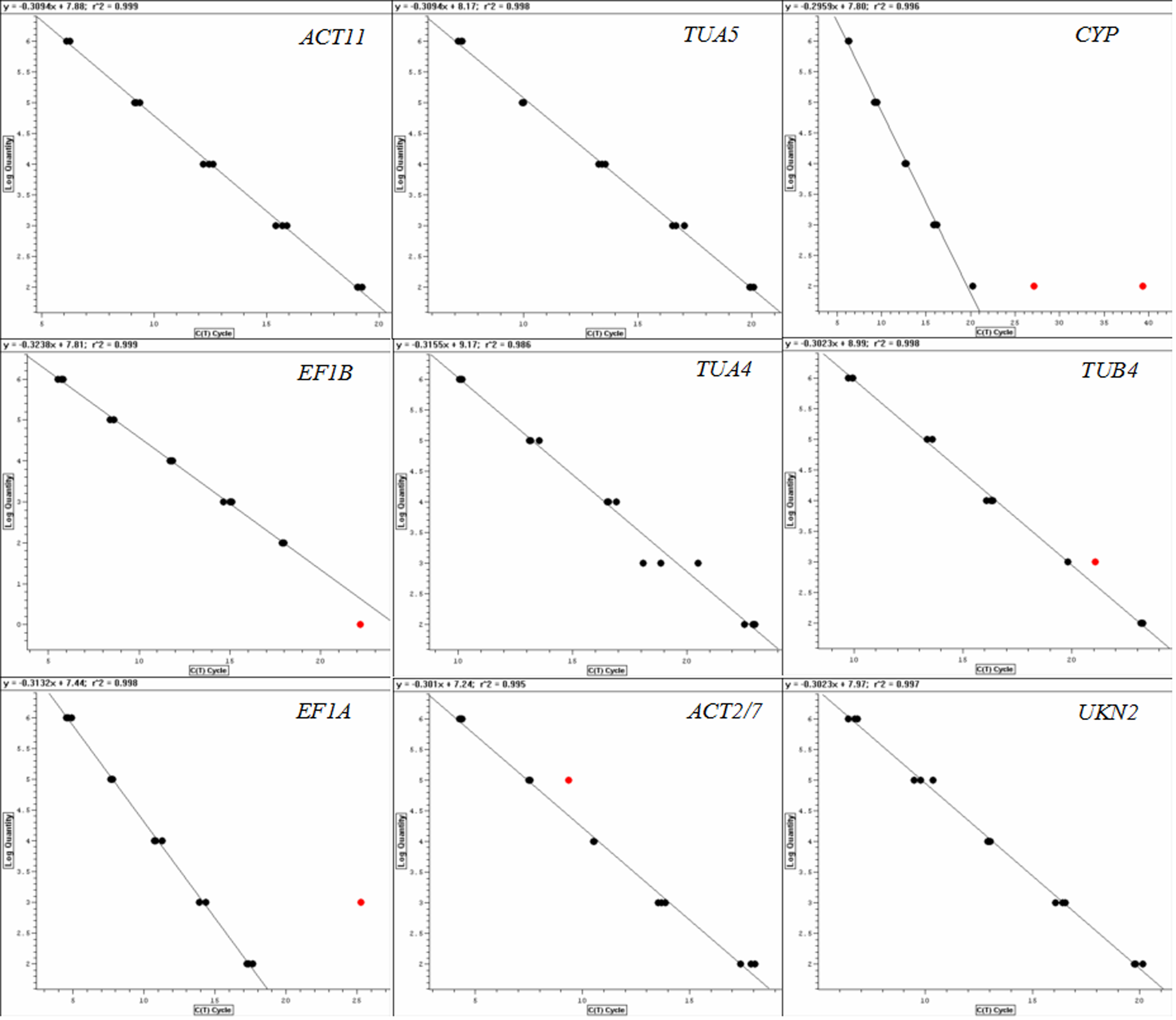

Supplement: Figure S3 — RT-qPCR standard curve of the 9 reference genes. (TIF) [file pone.0075271.s003.tif]
